# Transition in successors’ behavior and mindset while managing long-lived small and medium-sized manufacturing enterprises: a qualitative study

**DOI:** 10.12688/f1000research.52226.1

**Published:** 2021-04-12

**Authors:** Hiroo Suzuki, Yasunobu Kino

**Affiliations:** 1 Faculty of Business Science, University of Tsukuba, Tokyo, Japan

**Keywords:** Succession, successor, family business, long-lived SMEs, qualitative study, grounded theory approach, M-GTA

## Abstract

**Background:** There have been many studies conducted on succession, which can be considered as the most important issue in family businesses. However, most of these previous studies have focused only on the early stage of succession, uncovering the role of the predecessor and the successor. Only a few studies have made efforts to examine the total lifecycle of succession. The purpose of this study is to explore the process of the transition in successors' behavior and mindset while managing long-lived small and medium-sized manufacturing enterprises throughout the lifecycle of succession.

**Methods:** Semi-structured interviews were conducted with six successors of small and medium-sized manufacturing companies who are more than half a century old. Their answers were analyzed using the Modified-GTA method to construct a hypothetical model.

**Results:** In total, 46 concepts, four categories, 17 subcategories, and one core category were generated. An analysis result diagram using all concepts and categories was formed. From the observation of this diagram, the successors gained confidence in management through the dilemma between autonomy and constraint in the early stage of succession, which was found in previous research. Following the initial stage, the successors responded to the crisis caused by market constraints and created autonomous strategies in their businesses.

**Conclusions: **By experiencing repetitive crises, the successors tend to acquire new perspectives toward the naturally occurring crises. This change of premise by the successors is considered as the process of double-loop learning. Relationships inside and outside the company influence the generation of this viewpoint. From a long-term perspective, a sense of unity with employees, stable employment, and the pursuit of enjoyment constitute the successors' own values in this model.

## Introduction

Japan has more old companies than any other developed nation in the world. The number of centuries-old companies in Japan is 3,937, which is almost double than that of Germany, which has the second largest number of centuries-old companies with 1,850 ( The Nikkei, 2012). According to the database of a research company, there were around 33,000 centenarian companies in 2018 ( Teikoku Databank, 2019). Almost all of these companies are family owned, with 80.5% of them having net worth values that are below one billion yen. This means that the old companies in Japan comprise small or medium-sized family businesses.

Goto (2014) is known to have collected century-old company data on a worldwide basis. As per his findings, around 40% of century-old companies in Italy experienced ownership transfer to unrelated families. With respect to Switzerland, Goto (2014) found 56% of family-owned households to have experienced ownership transfers to another family. However, the transfer of ownership is extremely rare in century-old Japanese companies. This action is taken as the last resort to survive. By drawing from a comparison of countries via the worldwide survey, the traits of Japanese long-lived businesses have been family-owned.

Recently, small and medium-sized enterprises (SMEs) have become a serious issue with their succession of management. This tendency can be found from the symptoms of increasing aged-management and declining retiring ratio. While the average ratio of retiring CEOs was around 5% in the 1970s, it was 2.46% in 2011 ( The Small and Medium Enterprise Agency, 2016). The average age of the CEOs of companies is steadily increasing, and has currently reached 59.9 years old in 2019, the oldest ever recorded ( Teikoku Databank, 2020).

With the difficulty of succession in SMEs, the number of suspension firms is increasing ( The Small and Medium Enterprise Agency, 2019). The Japanese government is concerned that this may lead to the loss of employment and GDP in Japan in the future. Another survey ( The Small and Medium Enterprise Agency, 2016) shows that around half of the CEOs over 60 years are planning to abandon their businesses. Further, 44.9% consider their businesses to have at least 10 years of future possibility. As a result, the reason for the suspension is not the business itself, but the issue of the successor. From this viewpoint, one of the issues concerning the basic longevity of Japanese SMEs is the difficulty in tackling the succession problem.

The study of family businesses does not have a long history. In the mid-90s, the researchers of Harvard Business School focused on the uniqueness of family business and started studies in both U.S. and European business schools. At the same time, while Japan has a stronghold as the “family business nation,” the study of family business in Japan has a relatively short history ( Okumura, 2015). One of the reasons might be the overlapping of “ownership” and “management,” which is recognized as an obsolete style. The closed nature of family business might also be a reason for the lack of public information. This study attempts to explore the process of successors’ progress in long-lived SMEs to help the current CEOs and potential successors handle succession smoothly, which is the most important issue for SMEs.

### Theory and literature

*Three-circle model*

The modern form of the company is based on the separation of “ownership” and “management,” which has been advocated by Berle and Means (1932). Many business theories are based on this idea, such as agency theory, which illustrates that the adjustment of conflict between a company’s owner and manager is the main issue of company governance.

With respect to the case of family business, Tagiuri and Davis (1982) presented the “Three-circle model”, which explains the crossover of “Ownership,” “Family” and “Business” as an essential aspect of family business, and claims family business decision making to be determined by balancing the interests of three stakeholders.

*The 4Cs model*

Miller and Le Breton-Miller (2005) researched the factors for successful family-controlled businesses by comparing 46 successful and 24 struggling family-controlled businesses. Successful factors founded from their qualitative study are concluded as “the 4Cs”: continuity, community, connections, and command. A brief explanation of these factors is provided in Table 1. Okumura (2015) is of the opinion that the balancing of these factors is important for family business operations.

**Table 1.** The 4Cs of successful family-controlled businesses (based on [ Miller & Le Breton-Miller, 2012]).

The 4Cs	Priorities
Continuity	Pursuing the dream: pursue an enduring and substantive mission.
Community	Uniting the tribe: nurture a caring culture, made up of motivated people.
Connection	Being good partners: develop lasting and win-win relations with the outside.
Command	Acting and adapting freely: courageous and adaptive decisions.

*Empirical studies on family business*

Asaba (2015) also summarized previous studies on family businesses. In the early stage, the researcher conducted a direct comparison of the performance of family-owned and non-family firms. However, the superiority or inferiority of family businesses pertaining to performance is unclear from the standpoint of Japanese cases ( Saito, 2008; Mehrotra et al., 2013) as well as worldwide cases ( Anderson & Reeb, 2003). Thus, the interest of studies has shifted from benefit to behavioral characteristics such as investment or market strategies.

Having examined the research on investment in family and non-family businesses, Asaba (2013) concluded that family businesses continued their investment under volatile and stagnant economic conditions, which is difficult for non-family businesses. From the R&D investment viewpoint, family businesses are “avoiding radical innovations and pursuing incremental innovations” ( Asaba & Wada, 2019, p.277). These studies imply that family businesses have long time horizons and consider continuity, which is one of the traits of their strategy.

*Succession of family businesses*

Chrisman et al. (2003) examined papers on family business management from 1996 to 2003. They found 29 of the 190 strategy-related papers, 22.1% of the total, that were related to the succession of companies (Category 1). The other categories include economic performance (15.3%) and corporate governance (9.5%). This research study brings to light the fact that succession constitutes a major interest among researchers. Having reviewed worldwide studies related to family business succession, Iguchi (2019) found that there are three major research questions: 1) which factors affect the choice of the successor of a family business; 2) which factors affect the success or failure of a family; and 3) how much does succession impact the performance of the family business. These questions were derived from the top 50 cited papers on economics and management. Iguchi (2019) concluded that the impact on performance, including financial analysis should be studied as empirical studies (research question 3). However, previous studies have only covered the factors responsible for the selection of successors (research question 1), and the factors responsible for the success or failure of succession (research question 2), which were were mainly derived from case studies. The many parameters of the family business, such as family connections or emotional factors make empirical studies on these fields difficult, the exception being performance analysis. Thus, the accumulation of case studies is important for the maturation of this field of study. Iguchi (2020) extended his study to find the correlation between the preparation of the successor and the intention for long-term investment by the current CEO. This is fruitful research, as it should that the CEO’s current decision-making affects the expectations of his/her successors.

Kamiya (2018) defined these five factors as the unique issue of SME management and innovation, and created an analytical framework by combining these five items with the timeframe, starting from “Unfreeze,” to “Change,” and then to “Freeze,” introduced previously by Lewin (1947). By interviewing the management of a certain food company, Kamiya (2019) presented the matrix of the five factors of SMEs and Lewin’s three-step transition (unfreeze, change, freeze) as the process illustration of SME transformation.

*Modeling of the succession process*

Le Breton-Miller et al. (2004) created a diagram of an “integrative model of the succession process” by combining empirical research, theoretical research, and anecdotal evidence. In particular, they demonstrated the industrial context and social context, which was previously neglected in the inner-process of the family business. Moreover, a broader viewpoint is required to model the succession process of family businesses. In this study, in order for the transition in family business to be complete and smooth, not just the factors within the family or company, but also industry and social factors should be considered.

Ochiai (2016b) presented the idea of the successor’s dilemma between autonomy and constraints, which is drawn from the expansion of the observation of many case studies carried out by Gersick et al. (1997).

Previous studies have recognized that the successors from owners’ families are more likely to introduce discontinuous change than the internally promoted managers, for the successors from the owners’ families do not have to consider the predecessors and current employees ( Kagono, 2008). From this explanation, the successors seem to conduct autonomous activities. Ochiai stated that the successors from the owners’ families are ascribed status of being the future management and are treated as special person in the family-owned company. Ochiai further stated that they negotiate equally with the current president and have little concern about the employees. However, the successors are under constraints such as limited choice of occupation, restriction from the current management team, or psychological distance from the employees. The intention of reducing this dilemma is to establish the successors’ achieved status and gain acquired legitimacy as future CEOs with their innate legitimacy from the family.

Ochiai (2016a) also found a systematic approach to reduce this dilemma in well-established family-owned companies by conducting interviews with both the current CEOs and the successors from several well-established Japanese companies. This resulted in the formulation of the diagram “autonomy under guardianship”. This diagram presents the current CEO’s role as that of a guardian who gives proper assignments to the successor in order for him/her to adjust to their autonomous behavior and acquire legitimacy. Such modeling of the successor’s behavior is a unique study to understand the wisdom of a well-established family business.

### Study purpose

The duration of CEOs in a family-owned company is generally 20 years long, for it has been proven that the family-owned businesses’ peak profits and growth usually come at the end of their long tenure ( Yamaguchi, 2014; Miller & Le Breton-Miller, 2005). However, many previous studies operated within the framework of business succession, and the research that ended during the period of succession had been completed for several years. Only a few studies have brought out the complete lifecycles of successors, right until the end of their tenure as top management. Ochiai (2017) also stated that current studies focused only on the transition process itself, such as the analysis of the early stage of succession or the transition process of president influence, not covering the entrepreneurship of the successors during the middle of their managerial experience.

The purpose of this study is to try and find the process of succession in long-established family-owned SMEs and understand how successors established their mindsets as the owners of companies and top management post the early stage of succession. The modeling of these processes might be of help to potential successors during their preparation for a smooth succession.

## Methods

### Study design and participants

From April to August 2020, semi-structured interviews were conducted with the management of six companies. The interviewees were selected by purposive sampling to meet the following criteria: pre-, current, and former CEOs of family-owned manufacturing SMEs that are more than 50 years old and have been managed by three generations. Methods of approach for participants were face to face, email, telephone, or online. The list of interviewees with their attributes and the contact measures for participation is shown in Table 2.

**Table 2.** The interviewees included in the study.

Interviewees (approach to participate)	Business domain	Business model	Company history	Interviewee title (age)	Interview duration and type
Interviewee A (face to face)	Uniforms	business to consumer	120 years	Former CEO (70s)	100 min face to face
Interviewee B (email)	Cosmetics	business to consumer	120 years	Vice president (30s)	60 min online
Interviewee C (email)	Printing	business to business	80 years	President (60s)	75 min online
Interviewee D (telephone)	Parts-milling	business to business	53 years	President (60s)	50 min face to face
Interviewee E (telephone)	Parts-milling	business to business	90 years	President (60s)	105 min face to face
Interviewee F (online)	Plating	business to business	100 years	Vice president (40s)	80 min online

### Ethical considerations

In accordance with the ethical guidelines of University of Tsukuba, Faculty of Business Science, concerning human beings or specimens, no ethical approval was sought for this study. This is due to the study not involving any physical or mental intervention on the participants. Written informed consent to participate was obtained from each participant prior to the interview.

### Data collection

The interview time ranged from a minimum of 50 minutes to a maximum of 100 minutes (average time was 78 minutes). All interviews were conducted at their workplace face-to-face or online without the presence of any other participants ( Table 2).

Prior to the interviews, the participants agreed on the purpose of the interview and the expected questions, and they granted permission to audio recording for research purposes.

The verbatim transcriptions were made from the entire recorded data so that the field notes were not created through the interviewing process. Transcripts were not returned to the participants.

The interview items are as follows: 1) self-introduction, 2) the business domain and the transition of the main business, 3) how to tackle the past market change, 4) how to access the new market, 5) the creed related to the organization, human resources, and management philosophy, 6) future perspectives on business, and 7) anything else.

### Data analysis

The data were analyzed using the modified grounded theory approach (M-GTA) ( Glaser & Strauss, 1967). The GTA is a qualitative study method to generate “grounded theory” based on actual data by creating “concepts” from the original data and finding the relationship between these concepts. One of the features of the original GTA is the process of fragmentation of sentences to create the concept by removing the researcher’s bias ( Saiki-Craighill, 2014). M-GTA is a modified version of the original grounded theory approach, which holds the intention of being rigorous and scientific with the symbolic methodology of fine fragmentation of sentences constituting as raw data for research. Kinoshita (2003), who developed the M-GTA method, noticed that the original GTA method lost the context, including the cultural aspect, and hindered the formulation of richer concepts from the data ( Kambaru, 2018). One of the key features of M-GTA is its avoidance of the fragmentation process by keeping the rigorousness of the original GTA method intact and introducing the procedure of creating the “concept,” which refers to the analytical theme and analytical person, by using an “analytical worksheet” to balance the deep interpretation of data and the rigorous procedure.

M-GTA is a tested method for finding the process in social human interaction and solving the actual problem via a generated theory ( Kinoshita, 2007); therefore, this method is judged to be appropriate for this study. The analytical theme of this study is finding the process of succession and developing the management principle in long-lived small and medium-sized manufacturing enterprises *.* The analytically focused persons are successors in long-lived family-owned manufacturing SMEs, up to some hundred employees with a history of more than a half-century.

The analytical procedure consists of the following steps based on Kinoshita (2003).

**Figure 1.**
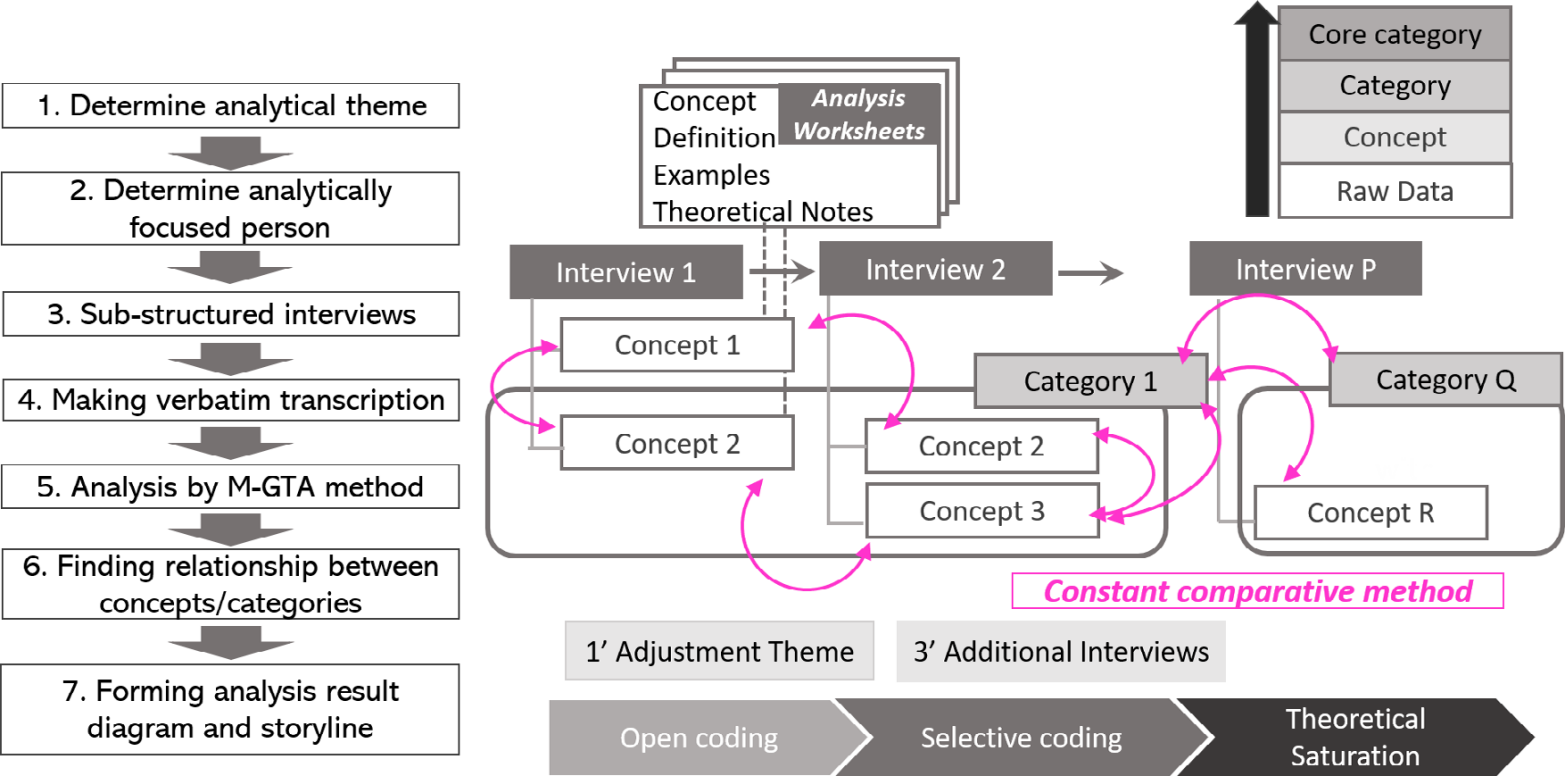
The procedure of the M-GTA method (modeled by the author).

1) Select the most detailed and richest transcription and select relevant parts of the statement from the selected transcription based on the analytical theme.
2) Determine the “concept name,” “definition” from “examples,” and the statement with “theoretical note” as the process of forming Analysis Worksheets, which is prepared for each concept.
3) During the process of determining the “definition” and “concept,” similar sentences from another transcription are selected and collected in the field of “examples” to prove the variety of sentences. Questions or ideas brought out from examining the sentences, but are not adopted in the definition, should be written in a theoretical note, and be confirmed and resolved during the progression of the analysis.
4) An important process while generating worksheets is to try and find not only “similar examples,” but also “polar examples” in the total data via concept generation to reduce the possibility of arbitrary interpretation. An example of an analytical worksheet in this study is presented in Table 3.
5) The generation of concepts is finished when no new concepts are distilled, which is considered to reach the “theoretical saturation.” In this study, the new concept was not generated during the analysis of the sixth interviewee so that the data saturation was considered to be achieved in this stage.
6) Examine the relationship of each concept, and create a “category,” which is the integrated idea of several concepts. The “analytical result diagram” showing the relationship between “categories” is formed as the result of the analysis. Figure 1 presents the overall analysis procedure for the M-GTA method.

**Table 3.** An example of an analytical worksheet created in this study.

**Concept name**	Acceptance of repetitive crisis
**Definition**	Reflecting the history, accepting the crisis should be repeated
**Examples**	Looking back on that, there were a lot of pinches that seemed to collapse. In the old days, the atomic bomb was dropped and we almost died, and after that, there was an oil shock, bubble burst, an IT bubble, Hanshin-Awaji earthquake, September 11, Lehman shock, East Japan earthquake, and so on. After all, it comes repeatedly. (Interviewee C)
	As I mentioned at the beginning, in about 10 years there has been a number of big things that happened, not just COVID-19, but Lehman shock, the bubble burst, 1st and 2nd oil shock, something big happened. It seems that we can flexibly respond to major changes in the industry among companies. After all, I think it is the robustness of the corporate constitution, and then we have to have strong finances. (Interviewee A)
**Theoretical notes**	CEOs are doing business under the premise that the good time and bad time of financial difficulty would happen, and which is the viewpoint of well-established companies and the successors who plan to manage for a long time.

## Results

**Figure 2.**
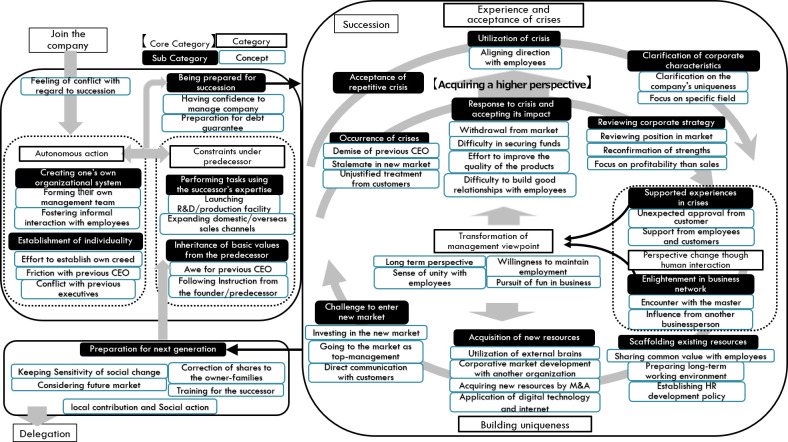
Analysis result diagram generated in this study.

As a result of the analysis, 46 concepts, 17 subcategories, four categories, and one core category were generated and reached theoretical saturation. These categories and concepts are listed in Table 4. The analysis result diagram integrated with these elements is shown in Figure 2.

**Table 4.** Extracted core category, subcategory, category, and concept with their definitions.

Core category	Category	Subcategory	Concept	Definition
	***Autonomous action***	*Establishment of individuality*	33: Effort to establish own creed	Effort to establish one’s own values ​​and ways of thinking as a successor
			8: Conflict with previous executives	Disagreement with the predecessor’s leader and executives
			34: Friction with previous CEO	Differences in values ​​and conflicts with previous management
		*Creating one’s own organizational system*	20: Forming their own management team	Creating a next-generation system, leading position, and training of right-handed people
			39: Fostering informal interaction with employees	Efforts to create an informal relationship with employees as a successor
	***Constraints under predecessor***	*Inheritance of basic values*	35: Following Instruction from the founder/predecessor	Lessons learned as the teachings of the preceding president or the founder
			36: Awe for previous CEO	Respect for the decisions and reputation of the predecessor
		*Performing tasks using the successor’s expertise*	31: Expanding domestic/overseas sales channels	Expanding sales channels in domestic and overseas market
			32: Launching R&D/production facility	Launch of resources such as R&D department and production department
		*Being prepared for succession*	37: Preparation for debt guarantee	Preparation to personally guarantee company debt
			38: Having confidence to manage company	Being confident in managing the company that was acquired after joining the company
			41: Feeling of conflict with regard to succession	Resistance to proactively taking over the business
**Acquiring a higher perspective**	***Experience and acceptance of crises***	*Occurrence of crisis*	3: Stalemate in new market	A newly tackled market gets stuck due to sluggish sales or lack of technology
			2: Demise of previous CEO	First recognition of lack of social credibility and psychological burden when the predecessor passed away
			1: Unjustified treatment from customers	Unjustified response from customers almost like subcontract bullying
		*Response to crisis and accepting its impact*	4: Effort to improve products quality	Trying to meet the high quality requirements compared to conventional products in the new market
			9: Difficulty in securing funds	Struggles to secure funds with financial institutions or families
			10: Withdrawal from market	Withdrawal from market when the future perspective becomes difficult
			11: Difficulty to build good relationships with employees	Failure to build relationships with employees during a company crisis
		*Reviewing corporate strategy*	46: Reconfirmation of strengths	Reviewing company’s strengths to use it to build business strategy
			5: Reviewing position in the market	Looking back on their position in times of crisis
			30: Focus on profitability than sales	Management that emphasizes profit margin, not sales
		*Acceptance of repetitive crises*		Management based on the premise that the crisis will be repeated by looking back on the past
		*Utilization of crisis*	27: Aligning direction with employees	Using the crisis as an opportunity to motivate employees.
		*Clarification on corporate characteristics*	40: Clarification on the company’s uniqueness	Pursue uniqueness for a differentiating strategy
			29: Focus on specific field	As SME with limited resources, narrowing down to business areas fitting that fit the company size
	***Perspective change through human interaction***	*Supported experiences in crises*	6: Support from employee and customers	Supported experiences from customers and employees in times of crisis
			7: Unexpected approval from customers	Unexpected approval from a major customer obtained through business.
		*Enlightenment in business network*	16: Encounter with the master	Encounter with a person who should be called a management teacher
			17: Influence from another businessperson	Encounter with a businessperson with new perspective
	***Transformation of management viewpoint***		1: Long term perspective	Management based on a long-term perspective having the ownership
			22:Willingness to maintain employment	Management view that they are the ones with employees
			23:Pursuit of fun in business	Be conscious of seeking fun in business
			47:Sense of unity with employees	The feeling that management and employees are one team
	***Building uniqueness***	*Scaffolding existing resources*	45: Establishing HR development policy	Establishing the concept of human resource development unique to SMEs
			18: Sharing common value with employees	Share principles with employees, such as work ethics and gratitude
			19: Preparing long-term working environment	Creating an environment where people can work if they are motivated even after retirement
		*Acquisition of new resources*	12: Acquiring new resources by M&A	Acquisition of resources through M&A
			13: Utilization of external brains	Utilization of external experts as consulting management
			14: Application of digital technology and internet	Utilization of digital processing and e-commerce in the Internet age
			15: Collaborative development market with another organization	Launching new markets or products in cooperation with the outside organizations
		*Challenge to enter new market*	24: Entering and investing to new market	Entering or investing in a different market
			25: Going to the market as the top-management	Top-management themselves are willing to open up new cu **s**tomers and new markets
			26: Direct communication with customers	A market approach that creates opportunities for direct contact with customers
		*Preparation for next generation*	28: Local contribution and social action	Incorporate local and social contribution perspective in the business.
			29: Training for the successor	Educating attitude as a successor to next successor (son, son-in-law)
			42: Being sensitive to social change	Having sensitivity to capture the new phenomena of social change
			43:Considering future markets	Thinking about new market initiatives in the future that may not happen immediately
			44: Consolidation of shares into the owner-family	Consolidation of diversified stocks as a successor issue of owner companies

The results are presented using the storyline or detailed explanations of the analytical result diagram shown in Figure 2 via the use of **core category**, ***category***, *sub-category*, and concept, with examples of the statement using quotes selected from the interview data.

The successors of long-lived manufacturing SMEs join the company sometimes by having the feeling of conflict with regard to succession. They experience a dilemma between ***constraints under the predecessor*** and ***autonomous action*** by accumulating their business experiences. This model is not directly the same as the “Autonomy Under Guardianship Model” ( Ochiai, 2016a), however, it can be regarded as a similar transitional process of succession with there being a conflict between constraint and autonomy ( Ochiai, 2016b).

### Constraints under the predecessor

During the first stage of joining the company, the successor starts *performing tasks using the successor’s expertise* and improves his/her business performance by carrying out tasks related to his/her educational background or experience before joining the company, such as expanding domestic/overseas sales channels and launching the R&D/production facility.

While gaining work experience by following the instructions of the founder, the successor inherits the basic philosophy and management spirit. Besides, he/she acquires the awe toward previous management by knowing the predecessors’ excellent decision making during the past business development and their deep relationship of trust with customers. This way, the *inheritance of the basic values from the predecessors* is observed by following the examples of the following statement:

*“Well, the most important creed for me is developing people. I told my employees the other day if there were people A, B, and C who made products using the same milling machine program, the appearance of products will be different, depending on each person. This idea was taught to me by my father, and it is the reality.” (Interviewee D).*

Another example is the idea of frugality taught by the predecessor. When sales were increasing due to the bubble economy, the (predecessor) often said: *“suppress the spending.”* Another thing that often remarked was *“when you make a profit, you should store it, you shouldn’t use it just because you made a profit.” (Interviewee A).*

### Autonomous action

At the same time, the successor’s unique movement against company constraints were also observed at the same time. Since the successor is a special person in the company, he/she makes efforts to foster informal interaction with employees to have a close relationship, such as having a meal with them or holding sports events after business hours. Also, the successor starts forming their own management team to gradually build people of the same generation who meet his/her values ​​as a management team for *creating their own organizational system.*

While accumulating management experiences, the successor often encounters friction with the previous CEO or conflict with previous executives, which is the opportunity to clarify his/her ideas. And his/her proactive effort to establish one’s own creed by joining a gathering of employers’ groups or summarizing one’s thoughts in the notebooks can be found at the same time as the process of the *establishment of individuality.*

The typical conflict between the previous CEO and previous executives is a different perspective toward business expansion. An example of a conflict with the pre-CEO case is as follows:

*“When changing the company name (from Family-name to the new brand name), I felt the process to be quite difficult because of my predecessor. He told me, ‘I have been this name since I was born … On the contrary, I am not that name when I was born (for the successor is the son-in-law). The impact of changing the company’s name to a general brand had a great impact on the market. Even when opposed, I have challenged various things.” (Interviewee A).*

Another case is that of a conflict with the executive who supported the previous CEO:

*“There was a person who was the executive supporting my father. He worked with me for two or three years after previous CEO passing away, However, we had to part ways, as there was a difference in opinion. I wanted to expand the business proactively, but he stated that our business should move steadily and gain money from customers as a result. Aggressive business style made eventual deficit. I was worried about such a different perspective for business.” (Interviewee D).*

From the examples of conflict mentioned above, it is clear that the successors tended to acquire business expansion in the first stage. At the same time, they deepened the confidence of management via the accumulated business experience and having the confidence to manage their companies. Successors sometimes successfully overcome the hardship or limitations confronted by the predecessors, and these experiences help the successors build their confidence. The preparation for debt guarantee is also seen as the determination of *being prepared for succession*.

### Experience and acceptance of crises: experience phase

For the successors who gained certain confidence by overcoming the difficulty of the company, which was difficult for the predecessor, it will be inevitable for them to face *new* crises that are represented by external factors such as economic recession or internal factors such as strategic failure, one of the turning points for the successors.

The typical *occurrence of crisis* for successors is the demise of the previous CEO, and the successors recognize that they possess a weak social status and a heavy responsibility. There are external crises ( stalemate in new market) caused by market changes, economic fluctuations, and technological shortages in the new markets. Or, the successors may face unjustified treatment from customers, such as unreasonable price reduction requests from customers, which can be seen as bullying. The successors can be overwhelmed by the harsh reality.

*“My predecessor suddenly disappeared in 1991 when he was 67 years old. I was 45 years old at that time. Overall, this period was severe. The previous president suddenly disappeared, and the bubble economy had burst and sales fell steadily, resulting in about half of the sales.” (Interviewee A).*

When they entered the new market, the effort to improve the quality of products is a countermeasure to tackle the crisis in the market. This is because the requirement from the customer is unexpectedly higher than the conventional customers. However, these experiences lead to an improvement in the company’s technological strength. However, in some cases, withdrawal from market is unavoidable because of the sudden decline in the market. In addition to such a market struggle, they also face difficulty in securing funds. Their assets should be collateral, or they face the challenge of the funding support being denied by their relatives. Another case of trouble is the difficulty in building good relationships with employees, such as letting go a trusted employee when the company is going through a difficult phase. These are the processes of *response to crisis and accepting its effect.*

After overcoming the crisis this way, they have an opportunity to reconsider customers and markets as a *reviewing corporate strategy*, such as reviewing the position in the market or focusing on profitability than sales. In addition, they use the experience of the crisis as an opportunity to learn about the reconfirmation of strengths of the company:

*“Do not produce products and services that are totally different from the conventional one. After all, our greatest strength is in (a certain domain). Even if we already have cutting-edge IT, even if we can use AI, I allow them to give the green light when this technology is related to our domain business.” (Interviewee C).*

In the above example, the successor coined original phrases to redefine their business domain again after the succession. Thus, having a crisis experience confirms their original or strongest business domain.

### Building uniqueness

After reviewing the strategy through the experience of the crises, the successors try to *scaffold the existing resources* to make future preparations for handling a crisis during a normal or peaceful occasion. They conduct institutional approaches such as establishing HR development policy or preparing long-term working environment, as well as share common values with employees about business ethics. The following example is the sharing of important values with employees as a company policy:

*“In fact, there are some occasions when the customers ask us to do a strange or unreasonable business activity, but every employee is expected to say we can not do that activity in order to set ethical boundaries. If we do something wrong, the company will collapse in the future.” (Interviewee C).*

The successors expand new resources through the business network and enhance conventional in-house resources. Typical examples are acquiring new resources by merger and acquisition (M&A), utilization of external brains pertaining to financial support or improvement of the productivity in daily routine, and application of digital technology and internet, such as in-house digitization and utilization of e-commerce. Additionally, there are cases where collaborative market development with another group is undertaken with public institutions such as universities or large branded companies. While some are successful, the others are only a trial. However, these activities are derived from the successors’ interests and uniqueness toward the process of *acquiring new resources.*

After preparing the resource-based matters, the successors *challenge to enter the new market* by investing in the new market or going to the market as top management. Further, they emphasize direct communication with customers to increase the opportunities to hear the customers’ voices directly, even if they had utilized the internet and e-commerce channels. One example is that of the successor often making unannounced visits to the potential customers:

*“The employees hesitate to do sales activity for a new customer without an appointment because it’s usual to be refused. However, in my case, there is a possibility of not being refused by the potential customer because I am the president. Even if I am refused, I think it is a poor story for this customer to lose a good opportunity and I do not have so much pride. Direct visits are also a good opportunity for finding the reality of the customer who has a good reputation, but I can sometimes notice that doing business with this customer is risky.” (Interviewee C).*

### Experience and acceptance of crises: acceptance phase

However, the challenges posed by the new markets do not always go well, the sales do not grow as expected, and there are ups and downs because of the business cycle. The term of the presidency is around four or six years in general; however, the management of family-owned businesses continues their role a rather long time, around a couple of decades. This is because they seem to have an *acceptance of repetitive crisis* **,** such as economic or natural disaster, and even have strategic failures as the preconditions of business cycles.

*“My father, my grandfather, as well as me, made many mistakes. And I have prepared myself assuming that these kinds of incidents would happen repeatedly, and manage to continue the business today.” (Interviewee C).*

After accepting the possibility of making failures or facing the market difficulties repeatedly, the successors not only respond to the crisis but also try *utilizing the crisis* so that their response aligns with the employees in the crisis to facilitate new market entry or improvement in the execution of daily activities.

For example, the successor experienced employees’ alignment for a new strategy after experiencing a couple of repetitive economic crises.

*“The bubble-economy burst and sales had fallen considerably. Then, when sales recovered slightly, another depression, the “Lehman shock” occurred. Therefore, I wanted to recover from this sales decline by entering a new field. When I decided to enter it, the employees did not show much resistance; on the contrary, everyone aligned with my idea.” (Interviewee A).*

Another successor appreciated the crisis time because a period of crisis can clarify the weakness of a company:

*“I am very grateful for this kind of crisis because I can see that our weaknesses were exposed and clarified, the weakness, which had been recognized as a strength.” (Interviewee C).*

Through the experience of the crisis, the successor will redefine the domain of the business and provide clarification on the company’s uniqueness to focus on the points of differentiation with the competitors. Besides, as a small and medium-sized enterprise with limited resources, the focus will be on a specific field, such as reviewing the variety of product and service lineups and limiting the sales’ geographical region. These are a part of the *clarification of corporate characteristics*, which is carried out during the last stage of tackling the crises.

### Acquiring a higher perspective (core category)

As shown in Figure 2, the process of ***experience and acceptance of crises*** consists of two branches, which explains the reason why the recognition of crisis changed from the first stage to the experienced stage. Based on the scenarios of facing and recovering from the crisis, they acquired the viewpoint that the occurrence of the crisis is regarded as a “precondition” of management. It is a change of perspective from a passive to proactive handling of the crisis. This is the most unique point of successors who are aware of their predecessors’ past hardships and their own difficult experiences. Therefore, this process was determined as the core category in this research.

### Perspective changes through human interactions

In Figure 2, the large loop between ***experience and acceptance of crisis*** and ***building uniqueness*** is shown. Between these processes, the successors experience human interactions inside or outside the company. This human interaction affects the mindset of the successors.

While overcoming such a crisis, they experienced conflicts with employees. However, at the same time, the remaining employees and long-term customers supported the critical situation. As a result, support from employee and customers lead to the *supported experience in crises*, or they may experience an unexpected approval from customers amidst a deadlock in the new market. Such an unexpected emotional experience might have a considerable influence on the feelings of the successors. One interviewee recalled the appreciation of the customers and employees who supported the company during the crisis:

*“Two factors made us survive. One is help from the customers. Perhaps there was a rumor among the customers that we would collapse. However, customers continued to trade, support, and purchase from our company. The other factor is employees. Of course, some employees quit when we were in a difficult situation. Still, we had 20 employees who had stayed back and done their best.” (Interviewee C).*

The successor may also be affected by the encounter with the master or influence from another businessperson in management. For example, the successor reflects on the employees’ perspectives by meeting an honorable business person who cares about their employees. Another successor who inherited the family business was also inspired by the encountered businessperson who started the business in his favorite field and applied a unique perspective in developing a new business. It is often found that such social interactions with people inside and outside the company lead to the experience of being *enlightened by human interaction* **.** In one case, the successor was inspired by a certain founder of the business based on his interest or hobby, which is opposite to doing the business seriously:

*“The businessperson who influenced me was Mr. A, who forced me to buy a motorcycle. When he participated in motorcycle racing, he asked me to come to an event in the U.S. At the same time, Mr. B joined an exhibition in the U.S. The teacher of the Toyota style may get angry, but that is not the world I am used to living in. Japanese tend to be serious, but that is a different world; it is a kind of an extension of their hobbies.” (Interviewee E).*

### Transformation of the management viewpoint

In the early stages of succession, the successors’ values were created in relationship with the preceding CEO. However, due to the experience of the crisis and the interaction with employees and customers via the business network, the successors’ new perspective on creations has come to be observed. As a typical example, the successors turned out to have long-term perspectives, have the intention to protect employees with the willingness to maintain employment, and uphold the element of pursuit of fun in business in the succeeded work or new business development to motivate themselves and employees. A successor did try to include enjoyment at the workplace even under the current crisis:

*“I’ve been saying lately that we’ve been in the job for at least eight hours, and I don’t even see my partner’s face for eight hours. Working hours is the longest time in daily life and we cannot stay for eight hours in the workplace. If we feel sad at the workplace, then why do we all not create it that makes us happy and excited? I have been saying this since the pandemic started.” (Interviewee D).*

Other successors emphasized that the important factors for family business are unity and a long-term viewpoint:

*“For a major company, the term of the presidency is generally four or five years. However, since I have been the president for 20 years, I can look after the business for a long time, and I do not have to worry about the stock price.” (Interviewee E).*

As a long-existing company, *preparing for the next generation* is also an important theme. To stabilize the management as an owner company, training provided to the CEO’s successor or consolidation of shares in the owner-family are important preparations to have sustainable management. One interviewee shared with their successor the grave reality to help him/her understand the company’s total financial responsibility:

*“It is not special that the company may collapse and our own house may disappear. I realized that it is also natural that everything will disappear and I always keep such possibilities in mind, and I am telling the same thing to my son (who is the next successor). I also said to my son that there is no meaning in possessing the house, for such an asset will be taken away when the company will bankrupt.” (Interviewee C).*

On the other hand, the successors have been considering future markets by being sensitive to social change. Furthermore, they prepare business succession to the next generation by having a perspective based on corporate social responsibility such as local contribution and social action. Finally, they reconsider the *inheritance of basic values from the predecessor* again, and such basic values are passed on to the next generation.

## Discussion

In the early stages of the succession process, the dilemma of the successors, which is described by Ochiai (2016b), is observed by the concepts ‘ following the instruction of the founder/predecessor’ and ‘ conflicting with the previous CEO’. However, strict frameworks such as the “Autonomy Under Guardianship” model ( Ochiai, 2016a), which is defined as “autonomy with intergenerational conflict, restraint, and discipline acting as constraints and guardianship from the preceding CEO” have not been clearly observed in this study. One reason might be that the research subject of Ochiai’s study was large companies, in contrast to the companies in this research, which assign several divisions to the successors periodically. Some successors in this study joined the company to help the predecessors overcome hardship when the market was shrinking or the economy was declining; thus, there was little room to prepare for a gradual succession process. The early stage of the succession process is not simple in order to bring out the successors’ proactive behavior. However, the dilemma might be the inevitable process for overcoming the conflict and creating their axis and confidence as businesspersons, as well as accumulating legitimacy as successors.

In traditional crisis management theory, the next crisis is averted by elemental preparations through a single feedback loop ( Pearson & Mitroff, 1993). However, on real occasions, such preparations do not work well because the crisis has a dynamic framework that ensures that the static and elemental reduction analysis of crisis does not clarify the mechanism of the occasion and address the crisis; thus, the approach of double-loop learning is required ( Maenaka et al., 2006).

In the initial stage of formulating the analysis result diagram in this study, the crises were modeled as repeating cycles. However, as the analysis progressed, the successors seemed to change their premise of crisis, that is, the occurrence of crises and failures is a common issue for experienced successors. This can be interpreted as higher-order learning represented by double-loop learning ( Argyris & Schon, 1974). Namely, the single loop learning in this study is the preparation of countermeasures for the crisis. However, the successors change their assumption from preparing the crisis to accepting and utilizing the crisis. This can be interpreted as double-loop learning.

This means that preparing the countermeasures learned from the crisis is a part of the preparation **.** For a potential crisis, however, building a firm relationship that is supported by both the customers and employees might be supplemental, but an essential preparation to make a sustainable organization.

**Figure 3.**
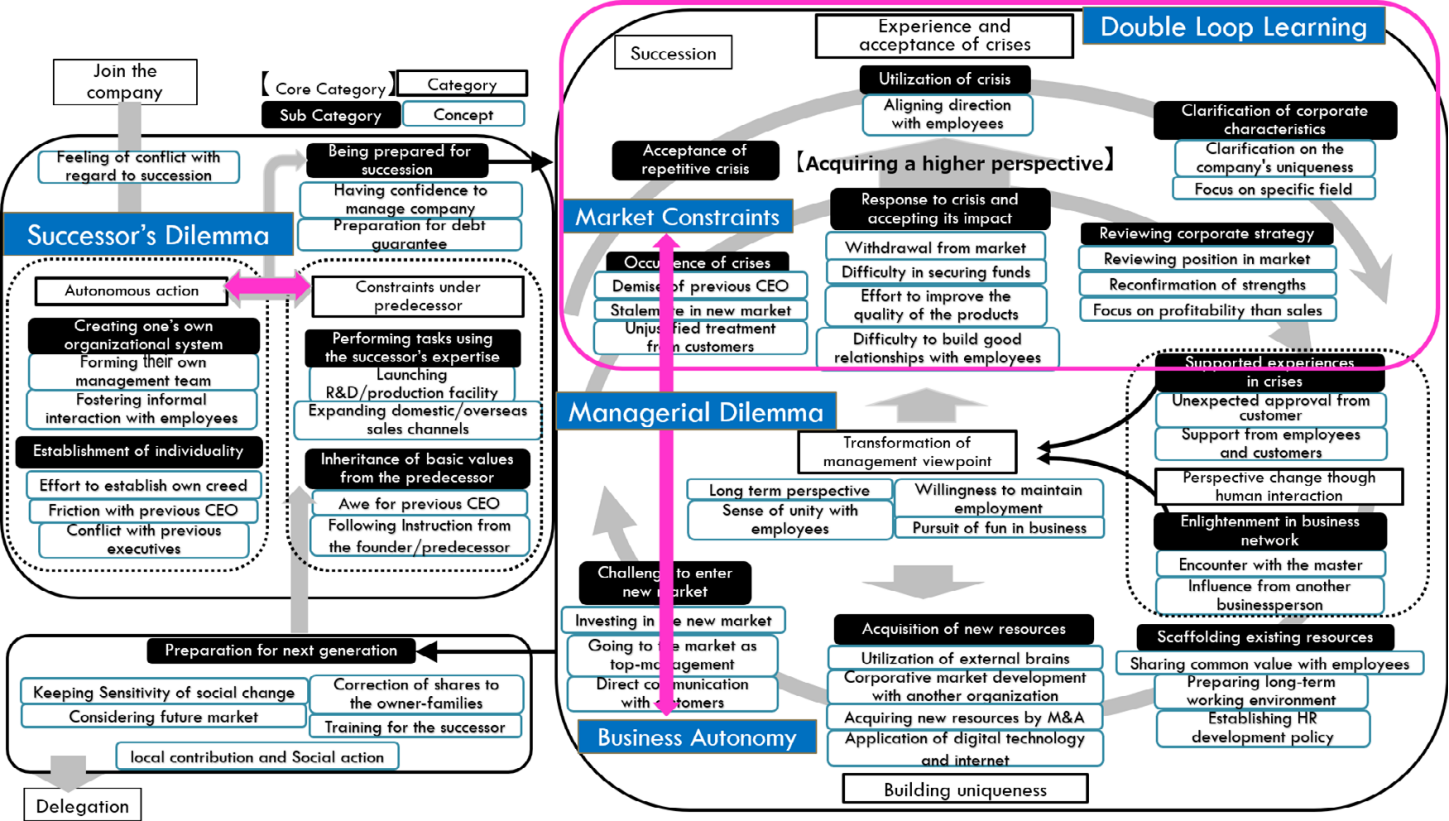
Interpretation of the analysis result diagram.

The part of the crisis that is caused by the limitation of the resources of the company such as technology or organization capability, which is functioning as the “constraint in the market,” and the process of building uniqueness is recognized as “business autonomy.” From this interpretation, the dilemma between “constraint” and “autonomy” does not conclude in the early stage of the succession process but continues throughout the entire business practice. It might be said that the dilemma of family business successors with regard to their predecessors is the preparation for the dilemma in their entire business lifecycle. These arguments are presented in Figure 3.

As mentioned in the section *Theory and literature*, the 4Cs theory is a collection of four priorities in strategies, organizational, and leadership priorities in family-owned companies derived from qualitative research on successful family businesses carried out by Miller and Le Breton-Miller (2005). The corresponding concepts in this study for these 4Cs elements are the following: continuity is a long-term perspective; community is the fostering of informal interaction with employees, sharing common values with employees, or having a sense of unity with employees; connection is corporative market development with another organization or local contribution and social action; and command is investing in a new market or going to the market as top management. The relationship between the 4Cs in the family business and the experience of crises have not been clearly described in previous research. However, the results of this study suggest that the repetitive crisis experience might affect the creation of such unique initiatives in family business.

### Limitations of the study

Our model is created based on the interviews with six persons, which is the methodological limitation of M-GTA. As a result, one should be cautious of making generalizations. Another limitation is the lack of comparison with other categories such as the manager of startups and the successor in service sectors, or another country case to clarify the uniqueness of the long-lived small and medium-sized manufacturing family businesses. This is also a limitation of M-GTA, which is focused on a specific group as an analytically focused person.

## Conclusions

The dilemma of the successors can be observed in long-existing family-owned companies, as previously described in the literature. However, this dilemma is considered to decrease as soon as successors have acquired legitimacy. In this study, the successors continue to experience dilemmas, even after taking over the presidency from the predecessors, by facing market constraints such as crises, and having autonomy in business creation. During business practice, the process of building perspective, right from tackling crises to accepting crises, is one development process of successors. Through these experiences, they formulate the principle of management, such as a long-term perspective or a sense of unity with employees. These perspective changes are also affected by the double-loop learning during crises and human interaction both inside and outside the company.

## Data availability

### Underlying data

The interviews were conducted under the agreement of keeping the participants anonymous and their interviews private. Therefore, the raw transcript data are not openly available. Quotes in the manuscript provide intermediate data. Transcripts will be reserved for at least five years; any interested readers can contact the corresponding author ( suzuki.hiroo.s5@nifty.com) to facilitate data access. Data will be shared under the following conditions: bona fide researchers with a proposal for how they wish to use the data.
