# Supplementary material for: Exploring the growth process of successors in long-lived small and medium-sized manufacturing companies: a qualitative study
Source: F1000Res. 2021 Aug 13;10:285. Originally published 2021 Apr 12. [Version 3] doi: 10.12688/f1000research.52226.3 (PMC8378407; doi:10.12688/f1000research.52226.3)
Supplement: Peer review history

# Peer review: Reviewer response for version 3

**DOI:** 10.5256/f1000research.59135.r91898

**Published:** 2021-09-13

**Authors:** Yi Zhu

**Affiliations:** 1 International Teaching Fellow, Lancaster University, Lancashire, UK

I am happy with the revised version.

Is the work clearly and accurately presented and does it cite the current literature?

Partly

If applicable, is the statistical analysis and its interpretation appropriate?

Not applicable

Are all the source data underlying the results available to ensure full reproducibility?

Partly

Is the study design appropriate and is the work technically sound?

Yes

Are the conclusions drawn adequately supported by the results?

Partly

Are sufficient details of methods and analysis provided to allow replication by others?

Yes

Reviewer Expertise:

Organizational management

I confirm that I have read this submission and believe that I have an appropriate level of expertise to confirm that it is of an acceptable scientific standard.

# Peer review: Reviewer response for version 3

**DOI:** 10.5256/f1000research.59135.r91899

**Published:** 2021-08-19

**Authors:** Chika Yoshida

**Affiliations:** 1 Faculty of Information and Communications, Bunkyo University, Shonan, Japan

I confirmed that the author has revised the paper in response to my respective comments and made it into the revised version 3.

 In addition, I did not comment regarding to the Method (M-GTA), but I was concerned that I might not be able to obtain enough data due to the small number of interviewees (6).

 However, I was able to understand the following statement in Data Analysis 5).

 "In this study, the new concept was not generated during the analysis of the sixth interviewee so that the data saturation was considered to be achieved in this stage in this stage."

 So, I do not see any problem with these data for the analysis. Therefore, I would like to rate this paper as "Approved".

Is the work clearly and accurately presented and does it cite the current literature?

Partly

If applicable, is the statistical analysis and its interpretation appropriate?

Not applicable

Are all the source data underlying the results available to ensure full reproducibility?

Partly

Is the study design appropriate and is the work technically sound?

Yes

Are the conclusions drawn adequately supported by the results?

Partly

Are sufficient details of methods and analysis provided to allow replication by others?

Partly

Reviewer Expertise:

Software Engineering (Process Model )/ Agile Development

I confirm that I have read this submission and believe that I have an appropriate level of expertise to confirm that it is of an acceptable scientific standard.

# Peer review: Reviewer response for version 2

**DOI:** 10.5256/f1000research.56341.r86529

**Published:** 2021-06-08

**Authors:** Yi Zhu

**Affiliations:** 1 International Teaching Fellow, Lancaster University, Lancashire, UK

I am happy with the revision. Good work.

Is the work clearly and accurately presented and does it cite the current literature?

Partly

If applicable, is the statistical analysis and its interpretation appropriate?

Not applicable

Are all the source data underlying the results available to ensure full reproducibility?

Partly

Is the study design appropriate and is the work technically sound?

Yes

Are the conclusions drawn adequately supported by the results?

Partly

Are sufficient details of methods and analysis provided to allow replication by others?

Yes

Reviewer Expertise:

Organizational management

I confirm that I have read this submission and believe that I have an appropriate level of expertise to confirm that it is of an acceptable scientific standard.

# Peer review: Reviewer response for version 1

**DOI:** 10.5256/f1000research.55472.r83250

**Published:** 2021-05-04

**Authors:** Chika Yoshida

**Affiliations:** 1 Faculty of Information and Communications, Bunkyo University, Shonan, Japan

Comments for each section:

**Abstract:**

- The purpose of this study is "to explore the process of the transition in successors' behavior and mindset," but it would be better to describe what you studied them for.
- As for the Conclusion, it is difficult to see not only what the common trends from the survey are, but also what they mean and how the results can be used.

**Introduction:**

- The data source in the third paragraph is a survey conducted in Japan, and it should be clearly stated as such.
- The second paragraph included examples from overseas, but the data source in the third paragraph is a survey conducted in Japan, and it should be clearly stated as such.
- The fifth paragraph describes the succession problem of family businesses. It would be better to indicate the direction of the study, such as whether this study can be used for that problem, or whether the data can be used to determine mergers and acquisitions like those of large companies, or succession outside the family.

**Theory and Literature:**

- In "Theory and literature," it is unclear whether the sections in that section are divided by model or category." For "Three-circle model" and "The 4Cs model," it would be better to title the section with what the research model is, so that it is consistent with other sections (such as "Empirical studies on family business").
- It is not clear the difference between this research and Ochiai (2016a).
- In the Discussion section, it is written that Ochiai (2016a) focuses on large corporations. That should be clarified in this chapter too.

**Study Purpose:**

- In the Abstract, you mentioned “The purpose of this study is to explore the process of the transition in successors' behavior and mindset while managing long-lived small and medium-sized manufacturing enterprises throughout the lifecycle of succession.” In the Introduction, you describe that “This study attempts to explore the process of successors’ progress in long-lived SMEs to help the current CEOs and potential successors handle succession smoothly, which is the most important issue for SMEs.”
  As with my comment for the Introduction, It would be better to indicate the direction of the study, such as whether this study can be used for that problem.

**Discussion:**

- There are some continuous descriptions for the previous studies, so it would be better to shift part of the discussion to the previous studies and summarize the discussion here.

**Conclusion:**

- There is a tendency to state all the issues of the study as limitations of the M-GTA method here. It is better to state how the issues of the M-GTA method will be resolved and studied in the future.

**General comments:**

- I have confirmed that this paper is of an acceptable scientific standard, but I would like the authors to revise the above comments and present the fundamental purpose of this research paper, how it can contribute to academia and what suggestions it can provide in practice.

Is the work clearly and accurately presented and does it cite the current literature?

Partly

If applicable, is the statistical analysis and its interpretation appropriate?

Not applicable

Are all the source data underlying the results available to ensure full reproducibility?

Partly

Is the study design appropriate and is the work technically sound?

Yes

Are the conclusions drawn adequately supported by the results?

Partly

Are sufficient details of methods and analysis provided to allow replication by others?

Partly

Reviewer Expertise:

Software Engineering, Requirement Engineering, Project Management

I confirm that I have read this submission and believe that I have an appropriate level of expertise to confirm that it is of an acceptable scientific standard, however I have significant reservations, as outlined above.

# Response

**Published:** 2021-08-01

**Authors:** Hiroo Suzuki

**Affiliations:** University of Tsukuba, Japan

We appreciate the comment by Prof.Yoshida and the following are the responses to each comment, which will be modified in Version 3.

*Abstract:*

*The purpose of this study is "to explore the process of the transition in successors' behavior and mindset," but it would be better to describe what you studied them for.*

 →Owing to the ambiguity of "behaviors and mindset", the research object described in the title and research purpose was changed to "exploring the growth process of successors.

*As for the Conclusion, it is difficult to see not only what the common trends from the survey are, but also what they mean and how the results can be used.*

 →In the discussion section, the academic and practical implication has been added.

*Introduction:*

*The data source in the third paragraph is a survey conducted in Japan, and it should be clearly stated as such.*

 →Stated.

*The second paragraph included examples from overseas, but the data source in the third paragraph is a survey conducted in Japan, and it should be clearly stated as such.*

 →Stated as the Japanese case in the third.

*The fifth paragraph describes the succession problem of family businesses. It would be better to indicate the direction of the study, such as whether this study can be used for that problem, or whether the data can be used to determine mergers and acquisitions like those of large companies, or succession outside the family.*

 →The purpose and benefits of the study were described from the perspectives of both the successors and the professionals who support them, along with the background of the problem of the business succession of SMEs in Japan and the government's measures to deal with it.

*Theory and Literature:*

*In "Theory and literature," it is unclear whether the sections in that section are divided by model or category." For "Three-circle model" and "The 4Cs model," it would be better to title the section with what the research model is, so that it is consistent with other sections (such as "Empirical studies on family business").*

 → The 4Cs model is categorized as "case studies" with another Japanese one.

*It is not clear the difference between this research and Ochiai (2016a).*

 →Ochai(2016a) is covering the period when the predecessors are in the top management, This study is following after the successors become the top. and different types of the dilemma is observed in early-stage and top management period, which are stated in the literature section and discussion.

*In the Discussion section, it is written that Ochiai (2016a) focuses on large corporations. That should be clarified in this chapter too.*

 →Reconsidering Ochiai(2016a), the main finding was the successors' transfer between several departments as the preparation of succession and does not clearly state the large company cases. And the uniqueness of Ochiai's study was interviewing both predecessors and successors in the same company so that the explanation of the context of his study has been amended.

*Study Purpose:*

*In the Abstract, you mentioned “The purpose of this study is to explore the process of the transition in successors' behavior and mindset while managing long-lived small and medium-sized manufacturing enterprises throughout the lifecycle of succession.” In the Introduction, you describe that “This study attempts to explore the process of successors’ progress in long-lived SMEs to help the current CEOs and potential successors handle succession smoothly, which is the most important issue for SMEs.”*

 →The study purpose is unified as exploring the growth process of successors in long-lived small and medium-sized manufacturing companies both

*As with my comment for the Introduction, It would be better to indicate the direction of the study, such as whether this study can be used for that problem.*

 →Adding the benefit of the study in the study purpose section.

*Discussion:*

*There are some continuous descriptions for the previous studies, so it would be better to shift part of the discussion to the previous studies and summarize the discussion here.*

 →Description of previous studies such as single and double-loop learning and crisis-management has shifted in the theory and literature part.

*Conclusion:*

*There is a tendency to state all the issues of the study as limitations of the M-GTA method here. It is better to state how the issues of the M-GTA method will be resolved and studied in the future.*

 →The necessity of further study based on a quantitative study in the part of the limitation of the study has been stated.

*General comments:*

*I have confirmed that this paper is of an acceptable scientific standard, but I would like the authors to revise the above comments and present the fundamental purpose of this research paper, how it can contribute to academia and what suggestions it can provide in practice.*

 →Study purpose with practical benefit has stated in the study purpose and implications.

# Peer review: Reviewer response for version 1

**DOI:** 10.5256/f1000research.55472.r83249

**Published:** 2021-04-27

**Authors:** Yi Zhu

**Affiliations:** 1 International Teaching Fellow, Lancaster University, Lancashire, UK

This paper uses modified GTA to explore the transition of successors at SMEs in Japan. It gives insights into the behavioral and mindset changes of the successor from a relatively long time span.

 My comments for each section as below:

**Abstract:**

- You may want to state the reason why the behavior and mindset of successors matter (the value of your research).
- Consider revising the title as it could be slightly long.
- To avoid misunderstanding, GTA may need a full name.
- Clarify what is the uniqueness of your research (some of the descriptions could be moved to the Conclusions).
- Need to explain further what type of crisis you refer to.

**Introduction:**

- Second paragraph: Isn't a Japanese company well known for having a successor from outside the family (i.e. 'mukoyoushi')?
- Third paragraph: you may want to specify which country/region you are referring to.
- Need discussion on the importance of SMEs in Japan (e.g. size of the economic loss).
- Unclear how the successor is an issue for the failing SMEs.
- 5th paragraph: the flow was unclear (how is short research life relates to the overlapping of ownership and management, could explore more on the latter topic).

**Theory and literature:**

- It seems too sudden to start this section: need introduction to the section.
- Need to clarify the relationship among these theories (e.g. how could 4Cs model relevant to the research by Asba?).
- *Succession of family businesses: *Which five factors that Kamiya is talking about?
- *Study purpose: *Could emphasize more on the uniqueness of this study which is to bring out the discussion on complete lifecycles of successors in Introduction and Abstract.
- Figure 2 is difficult to comprehend: could have two types of figures, one with a simpler contents; need clarification on the changes of the successors' behaviors and mindsets.
- Clarify how your research is different from Ochiai 2016a.
- Some codes are difficult to understand (e.g. what do you mean by 'Acquiring a higher perspective'?).
- More clarity on the transformation of the management viewpoint: discuss in early-stage in this section what kind of transformation you are referring to.

**Discussion:**

- Some early studies were only mentioned in this section, not in the literature review section, which may confuse the reader (e.g. single/double loop learning; crisis management).

**Conclusions:**

- Add the implications of the study.

Is the work clearly and accurately presented and does it cite the current literature?

Partly

If applicable, is the statistical analysis and its interpretation appropriate?

Not applicable

Are all the source data underlying the results available to ensure full reproducibility?

Partly

Is the study design appropriate and is the work technically sound?

Yes

Are the conclusions drawn adequately supported by the results?

Partly

Are sufficient details of methods and analysis provided to allow replication by others?

Yes

Reviewer Expertise:

Organizational management

I confirm that I have read this submission and believe that I have an appropriate level of expertise to confirm that it is of an acceptable scientific standard, however I have significant reservations, as outlined above.

# Response

**Published:** 2021-08-01

**Authors:** Hiroo Suzuki

**Affiliations:** University of Tsukuba, Japan

We appreciate the fruitful comment by Dr.Zoe, and the following are the responses to each comment.

*You may want to state the reason why the behavior and mindset of successors matter (the value of your research).*

 →Definition of "mindset and behavior" is vague so that changed the title to "growth process".

*Consider revising the title as it could be slightly long.*

 →Changed the title to the shorter one.

*To avoid misunderstanding, GTA may need a full name.*

 →Used full name instead of the abbreviation.

*Clarify what is the uniqueness of your research (some of the descriptions could be moved to the Conclusions).*

 →Described the difference and uniqueness of this study in literature review and conclusions.

*Need to explain further what type of crisis you refer to.*

 →market and customers issues have been described.

*Introduction:*

*Second paragraph: Isn't a Japanese company well known for having a successor from outside the family (i.e. 'mukoyoushi')?*

 →Described "mukoyoshi" or succession by sons-in-law, which is considered as the expansion

 of continuation of family-ownership in Japan.

*Third paragraph: you may want to specify which country/region you are referring to.*

 →Stated this is a Japanese case.

*Need discussion on the importance of SMEs in Japan (e.g. size of the economic loss).*

 →Stated the economic impact of the SMEs succession issue

*Unclear how the successor is an issue for the failing SMEs.*

 →Stated the economic impact of the SMEs succession issue

*5th paragraph: the flow was unclear (how is short research life relates to the overlapping of ownership and management, could explore more on the latter topic).*

 →The paragraph has replaced the explanation of the current government approach to tackle the succession issues as the background requires information and standardization of the succession process.

*Theory and literature:*

*It seems too sudden to start this section: need introduction to the section.*

 →The introduction part has been added.

*Need to clarify the relationship among these theories (e.g. how could 4Cs model relevant to the research by Asba?)*

 →Divided case studies part and other studies including Asaba's study.

*Succession of family businesses: Which five factors that Kamiya is talking about?*

 →Elaborated Kamiya's studies with five factors

*Study purpose: Could emphasize more on the uniqueness of this study which is to bring out the discussion on complete lifecycles of successors in Introduction and Abstract.*

 →Changed the study object to the “exploring the growth process of successors” and stated study purpose related to the object.

*Figure 2 is difficult to comprehend: could have two types of figures, one with a simpler contents; need clarification on the changes of the successors' behaviors and mindsets.*

 →Figure 3 was modified as the simpler version of Figure 2.

*Clarify how your research is different from Ochiai 2016a.*

 →Described the difference with Ochai2016a from the timeframe of the study.

*Some codes are difficult to understand (e.g. what do you mean by 'Acquiring a higher perspective'?).*

 →"Acquiring higher perspective" has changed to "Changing Perspective on crisis"

 "Transformation of management viewpoint" has changed to "Establishing management principles".

*More clarity on the transformation of the management viewpoint: discuss in early-stage in this section what kind of transformation you are referring to.*

 →The concept name has changed and recognized as the process of "establishing management principles".

*Discussion:*

*Some early studies were only mentioned in this section, not in the literature review section, which may confuse the reader (e.g. single/double loop learning; crisis management).*

 →Double-loop learning and Crisis management have been explained in the literature section with previous studies.

*Conclusions:*

*Add the implications of the study.*

 →Academic and practical implications were added.
